# Blood microsampling technologies: Innovations and applications in 2022

**DOI:** 10.1002/ansa.202300011

**Published:** 2023-05-18

**Authors:** Manchu Umarani Thangavelu, Bert Wouters, Alida Kindt, Irwin K. M. Reiss, Thomas Hankemeier

**Affiliations:** ^1^ Metabolomics and Analytics Centre Leiden University Leiden The Netherlands; ^2^ Department of Neonatal and Pediatric Intensive Care Division of Neonatology Erasmus MC Rotterdam The Netherlands

**Keywords:** dried blood spot, forensic toxicology, microsampling, therapeutic drug monitoring, volumetric absorptive microsampling

## Abstract

With the development of highly sensitive bioanalytical techniques, the volume of samples necessary for accurate analysis has reduced. Microsampling, the process of obtaining small amounts of blood, has thus gained popularity as it offers minimal‐invasiveness, reduced logistical costs and biohazard risks while simultaneously showing increased sample stability and a potential for the decentralization of the approach and at‐home self‐sampling. Although the benefits of microsampling have been recognised, its adoption in clinical practice has been slow. Several microsampling technologies and devices are currently available and employed in research studies for various biomedical applications. This review provides an overview of the state‐of‐the‐art in microsampling technology with a focus on the latest developments and advancements in the field of microsampling. Research published in the year 2022, including studies (i) developing strategies for the quantitation of analytes in microsamples and (ii) bridging and comparing the interchangeability between matrices and choice of technology for a given application, is reviewed to assess the advantages, challenges and limitations of the current state of microsampling. Successful implementation of microsampling in routine clinical care requires continued efforts for standardization and harmonization. Microsampling has been shown to facilitate data‐rich studies and a patient‐centric approach to healthcare and is foreseen to play a central role in the future digital revolution of healthcare through continuous monitoring to improve the quality of life.

**Abbreviations**
3Rs: Replace, reduce and refine
4OHCP: 4‐hydroxycyclophosphamide
BRP: Biological reference preparation
CE‐MS: Capillary electrophoresis‐mass spectrometry
CERA: Continuous erythropoietin receptor activator
CMS: Capillary microsampling
CP: Cyclophosphamide
DBS: Dried blood spot
DPS: Dried plasma spot
DSS: Dried serum spot
EDTA: Ethylenediaminetetraacetic acid
EPO: Erythropoietin
ERA: Erythropoietin receptor agonist
Hct: Haematocrit
HRMS: High‐resolution MS
IL‐1Ra: Interleukin‐1 receptor antagonist
IL‐1β: Interleukin‐1 beta
IMS: Immunosuppressants
LC: Liquid chromatography
mAb: Monoclonal antibody
MRPL: Minimum required performance level
MS: Mass spectrometry
MSW: Microsampling Wing
NESP: Novel erythropoiesis stimulating protein
NIR: Near infrared
NPS: New psychoactive substances
PD: Pharmacodynamic
pDBS: Patterned DBS
PEth: Phosphatidylethanol
PG: Prostaglandin
PK: Pharmacokinetic
PSI: Paper spray ionization
RBC: Red blood cell
SPE: Solid‐phase extraction
TD: Toxicodynamic
TDM: Therapeutic drug monitoring
Tepa: N,N'N’’‐triethylenephosphoramide
THC: Tetrahydrocannabinol
Thiotepa: Triethylenethiophosphoramide
TK: Toxicokinetic
TKI: Tyrosine kinase inhibitor
TMS: Tube microsampling
UHPLC: Ultra‐high‐performance LC
UPLC: Ultra‐performance LC
UV/VIS: Ultra‐violet/visible
VAMS: Volumetric absorptive microsampling®
VTM: Volumetric tip microsampling
WADA: World Anti‐Doping Agency

## 1 INTRODUCTION

Although invasive intravascular access has been the gold standard for blood sampling for decades and remains one of the most prevalent medical procedures in healthcare, its use is limited by several drawbacks.^1^ The process of acquiring intravascular access with a hypodermic needle for blood sample collection (typically > 1 mL) requires a qualified phlebotomist and a sterile clinical environment.^2^ This invasive and centralised approach is often associated with inconveniences such as frequent clinical visits, discomfort, anxiety, pain and phobia, which can lead to reduced patient compliance.^3^ Poor vascular puncture practices can result in haemoconcentration or haemolysis, rendering the samples unsuitable for analysis and subjecting patients to the inconvenience of a second blood draw. In addition, they expose healthcare personnel to the risk of sharp injuries and bloodborne pathogens. Occasionally, they may also lead to complications such as hematoma, infection, nerve damage and iatrogenic anaemia, potentially causing physical, psychological and economic distress.^3^, ^4^, ^5^, ^6^ Consequently, medical procedures may be delayed or disregarded, and participation in clinical research may be reduced.^7^ Furthermore, these samples necessitate time‐consuming, resource‐intensive and expensive sample collection and preparation protocols to minimise pre‐analytical variability.^8^ About 75% of samples require centrifugation to obtain plasma or serum for analysis, which increases economic costs and is a substantial factor for delays in a laboratory workflow.^9^ Wet blood samples require cold‐chain storage and shipping to prevent sample degradation and bacterial growth.^10^

Blood microsampling, the process of capturing small volumes of capillary blood (typically <100 µL), often in a minimally‐invasive manner, presents a viable alternative to vascular puncture and is a significant contributor in the revolutionization of human healthcare towards preventive, participative and personalised approaches in disease management. The minimal invasiveness of a low‐volume blood draw provides benefits over conventional blood sampling by enabling amenable self‐sampling without the need for trained personnel or hospital visits, which can advance primary healthcare in rural and remote geographical areas,^11^ and improve patient recruitment and retention, thereby increasing statistical power in clinical studies.^10^ Reduced blood volumes enhance the feasibility of sampling volume‐limited candidates and serial sampling, which can facilitate longitudinal investigations with fewer participants.^12^ In addition, dried microsamples eliminate the requirement for centrifugation and aliquoting, may be transported at ambient temperatures without compromising analyte stability, can facilitate direct processing and are compatible with automated workflows. Dried matrices also render most pathogens inactive, reducing the biohazard risk generally associated with the transfer of clinical samples.^13^ The enhanced feasibility of sample processing, storage and shipment significantly reduce the turnaround time of blood testing, hence promoting shorter time‐to‐diagnosis.^14^

The complexity of the blood matrix often necessitates elaborate sample clean‐up procedures such as protein precipitation, liquid–liquid extraction and solid‐phase extraction (SPE) to reduce matrix effects and enrich analytes to the desired detection limit.^15^ Since microsamples comprise only a few hundred microliters, highly sensitive bioanalytical assays are required for analysis in addition to improved sample clean‐up, particularly for low‐abundant target analytes. In addition to the conventional sample clean‐up procedures, microsample preparation has been enhanced by the development of new techniques such as porous polymeric thin‐film extraction,^16^ micro‐SPE with pipette tips and spin columns^17^ and three‐phase electroextraction, which is capable of achieving simultaneous clean‐up and high enrichment of 20 µL microsamples in a short amount of time.^18^ Automation of miniaturised extraction techniques and direct hyphenation to analytical instrumentation minimises sample loss and alleviates the sample‐preparation bottlenecks in volume‐limited samples.^19^ The most extensively employed quantitative analytical technique for microsample analysis is liquid chromatography (LC) separation coupled with mass spectrometry (MS) detection due to its ability to detect analytes in pg/mL concentrations. Other separation techniques such as capillary electrophoresis‐mass spectrometry (CE‐MS) provide a potent analytical tool for the efficient profiling of polar and charged analytes in small sample volumes via separation by an electric field.^20^ Furthermore, paper‐based ionization techniques such as paper spray ionization (PSI) for MS have enabled the direct analysis of a broad spectrum of analytes in dried microsamples without the requirement of any prior sample pretreatment.^21^ These sophisticated approaches have driven the implementation of microsampling in various biomedical domains; newborn and metabolic screening,^22^, ^23^ biomarker research,^21^, ^24^, ^25^ pharmacokinetic (PK) and pharmacodynamic (PD) studies,^26^, ^27^ therapeutic drug monitoring (TDM),^28^, ^29^, ^30^, ^31^, ^32^, ^33^ forensic toxicology,^34^, ^35^, ^36^ sports anti‐doping,^37^, ^38^, ^39^ metabolomics^22^, ^40^, ^41^ and proteomics.^24^, ^42^, ^43^ Since 70% of medical decisions are governed by diagnostic blood tests,^44^ several medical technology companies are investing in the development of innovative at‐home blood microsampling devices to make blood work more accessible and convenient. Being an integral part of the paradigm shift towards patient‐centric healthcare systems, the microsampling devices market is projected to reach a value of US$ 2.4 Bn by 2030 at a compound growth annual rate of ∼7%.^45^

**FIGURE 1:**
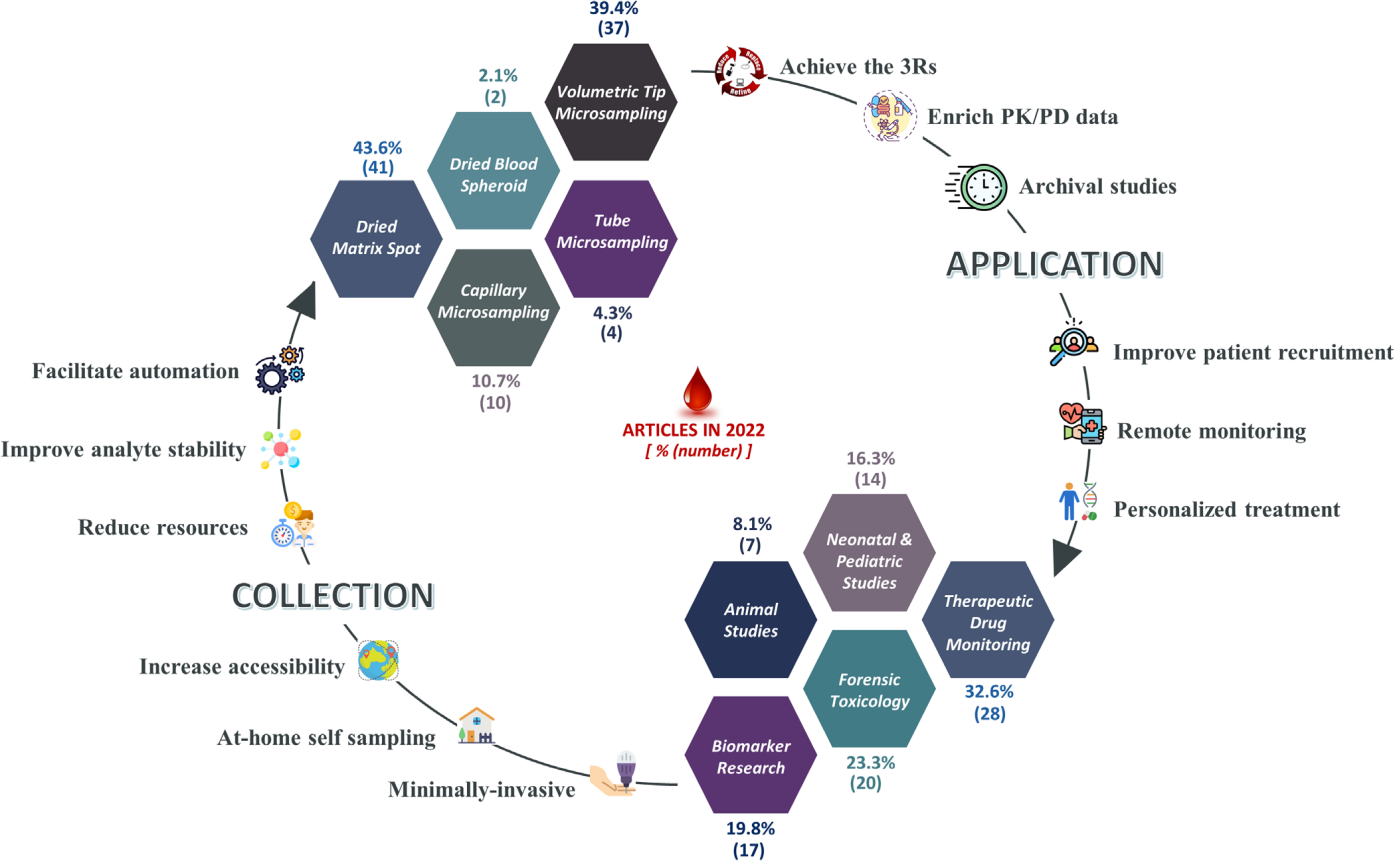
Graphical summary of the benefits of microsampling and 2022 publication statistics of microsampling technologies and applications.

This review provides an extensive overview of the state‐of‐the‐art in blood microsampling, with a focus on recent significant developments, technological advancements and applications, published in the year 2022. It includes a discussion on the working principles, advantages and challenges of various microsampling technologies and associated commercially‐available devices. The key developments in microsampling, which include (i) development and validation of bioanalytical methods for the quantification of analytes, (ii) validation of microsampling by comparison with conventional sampling with emphasis on interchangeability between matrices and subsequent determination of matrix conversion factors if needed and (iii) evaluation and comparison of the performance of two or more microsampling devices to determine the optimum for a given application, are summarised and reviewed. Figure 1 provides a visual summary of the benefits of microsampling and statistics of microsampling technology and applications in 2022.

## 2 MICROSAMPLING TECHNOLOGIES AND INNOVATIONS

Microsamples can be collected either as dried or liquid matrix microsamples. Existing technologies for collection of the former include the dried matrix spot, dried blood spheroid and volumetric tip microsampling. The latter can be collected either using a capillary‐based or tube‐based technology. The following sections provide a comprehensive overview of the microsampling technologies, including commercially‐available devices and recent innovative concepts for microsampling.

### 2.1 Dried matrix microsampling

An overview of commercially‐available dried matrix microsampling devices can be found in Table 1.

**TABLE 1:** Overview of commercially‐available dried matrix microsampling devices.

Technology	Classification	Sub‐classification	Device	Company	Design	Volume	Samples	Advantage	Disadvantage
Dried Matrix Spot	Dried Blood Spot	Non‐Volumetric Dried Blood Spot	Conventional Filter Cards (Whatman 903)	Cytvia (Massachusetts, United States)	rectangluar filter paper held by a sturdy card or plastic frame to provide rigidity and stability during handling and processing	∼80 μL	5	cost effective; easy to handle; compatibility with automated lab processing	potential volume bias; easy contamination of sample
			HemaSpot HD	Spot On Sciences (San Francisco, United States)	moisture‐tight cartridge incorporated with sampling membrane and dessicant	∼160 μL	Multiple punches	opportunity to run a variety of assays from a single sample; patented membrane said to reduce hematocrit effect	potential volume bias
		Volumetric Dried Blood Spot	HemaXis DB10	DBS Systems SA (Gland, Switzerland)	microfluidic chip with a panel of microfluidic channels and standard Whatman filter card encased in a protective plastic cassette	10 μL	4	compatible with automated lab‐processing systems	easy contamination of sample due to open device format; incomplete channel filling or transfer; incompatibility with blood containing organic solvents
			Capitainer B	Capitainer AB (Stockholm, Sweden)	microfluidic channels with inlet and outlet ports with dissolvable films	10 μL	2	eliminates risk of over‐ or under‐ filling by integrating successful sampling indicator	possibility of incomplete channel filling
			HemaPEN	Trajan Scientific and Medical (Melbourne, Australia)	K2 EDTA‐coated microcapillaries and pre‐punched DBS discs housed in a transparent housing and a base	2.74 μL	4	No sample contamination due to closed housing	requires manual labor and specialized hemaPEN opening tool to retrieve samples
			HemaSpot HF	Spot On Sciences (San Francisco, United States)	cartidge containing an application surface with small opening, an underlying absorbent fan‐shaped collection matrix with 8 blades, and dessicant	10 μL	8	eight replicates without the need for punching, allowing for repetitive, reproducible, and multiple testing; innovative HemaForm absorbent paper said to promote uniform distribution	possibility of incomplete blade filling
	Dried Plasma Spot		Telimmune Card	Telimmune (West Lafayette, Indiana, United States)	card format with spreading membrane, separation membrane, and collection discs	3 μL plasma from 25 μL whole blood	1 or 2	volumteric plasma collection from non‐volumetric whole blood application; plasma collection without centrifugation	
	Dried Serum Spot		HemaSpot SE	Spot On Sciences (San Francisco, United States)	cartridge containing spiral shaped membrane and dessicant		Multiple punches	Separation of whole blood into its constituents without centrigugation	
Volumetric Tip Microsampling			Mitra	Trajan Scientific and Medical (Melbourne, Australia)	plastic handler with a proprietary hydrophilic polymer tip	10 μL, 20 μL, or 30 μL	1	volumetric collection independent of hematocrit	possibility of background signal from the polymer tips; contamination onto plastic handler; Only one sample per tip‐multiple samples for testing; expensive
			TASSO‐M20	Tasso Inc. (Seattle, United States)	device with button, lancet, microfluidic channels connected to a sample pod with volumetric tips	17.5 μL	4	volumetric collection independent of hematocrit	possibility of background signal from the volumetric tips;

#### 2.1.1 Dried matrix spot: Dried blood spot, dried plasma spot and dried serum spot

The dried blood spot (DBS) is the oldest microsampling technique and is well established in developed countries for newborn screening of more than 50 inborn genetic and metabolic disorders, while gaining popularity in various other biomedical applications.^46^, ^47^, ^48^ DBS samples are capillary blood droplets – from a lancet prick on the finger, heel or toe – dripped onto specially manufactured filter paper comprising a sheet of thick cellulose cardstock affixed to an envelope for sample identification and handling. The saturated filter paper is dried at ambient temperature for a minimum of 4 h and shipped to a laboratory for analysis, where fixed‐diameter discs are punched out from the filter paper to provide volumetric defined measurements, and blood components are extracted by rehydrating the filter matrix with an elution buffer.^15^

**FIGURE 2:**
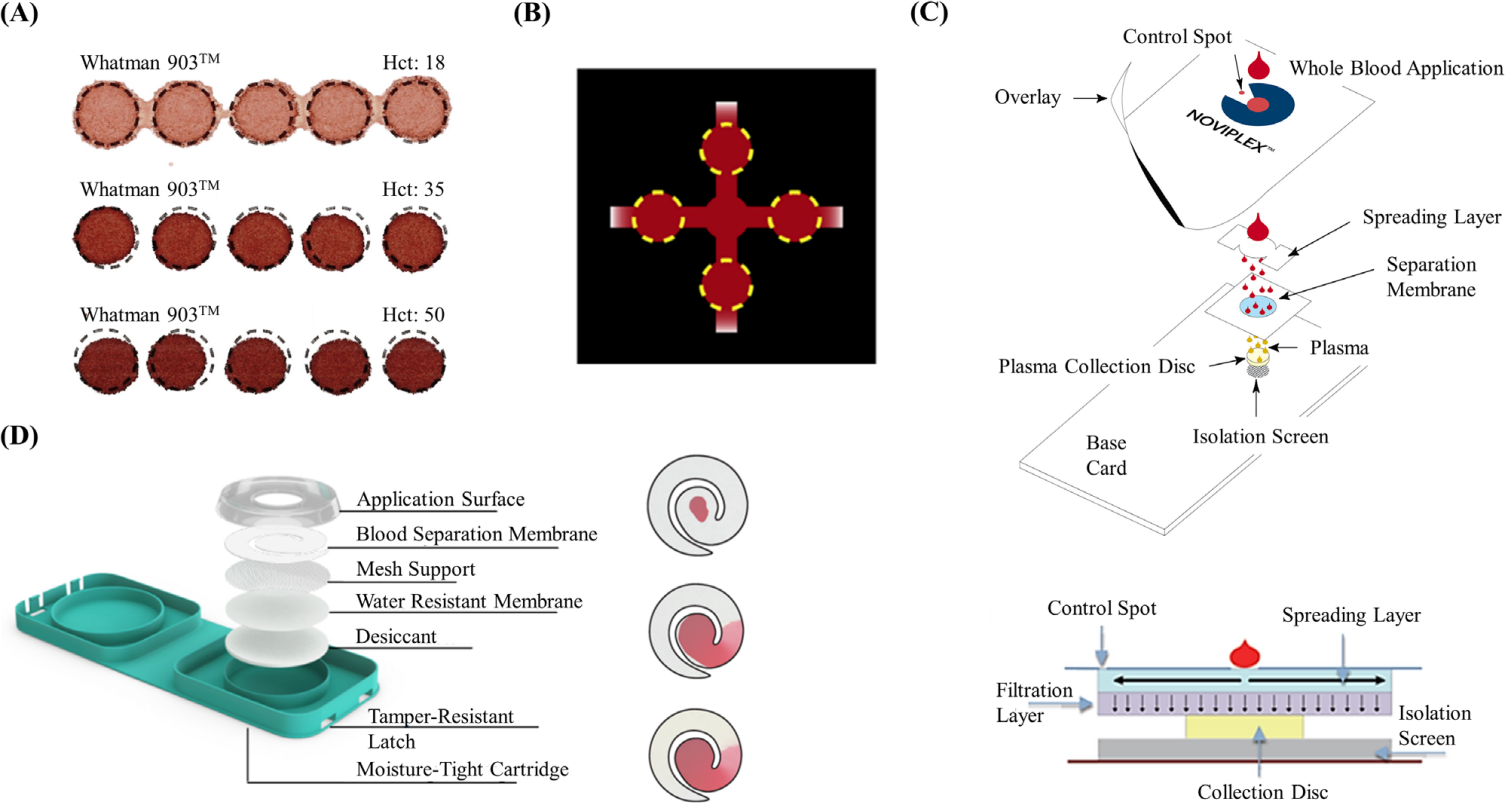
Dried matrix spot technology. (A) Dried blood spot at different levels of blood haematocrit. (B) Patterned dried blood spot with four uniform replicates. (C) Telimmune plasma separation card with cross‐sectional view. (D) HemaSpot SE device with spiral membrane to facilitate separation of whole blood into constituent parts. (Adapted from References 50, 56, 69 and 70)

Despite its simple blood collection process, DBS use in biomedical applications is limited by multiparametric sources of variability that can impact quantitative analytical accuracy.^13^ Haematocrit (Hct), that is, the volume proportion of red blood cells (RBCs) in blood is the predominant issue of DBS as it influences DBS spot size, spot homogeneity and extraction recovery of analytes.^49^ Blood with higher Hct levels spreads more slowly on filter paper due to increased blood viscosity, producing a smaller spot than blood with lower Hct levels for the same volume of blood (Figure 2A).^50^ This spot bias results in unreliable volumes in fixed‐diameter punches impacting the accuracy of analyte quantitations.^49^, ^51^ Further analytical biases are introduced by the inherent component of DBS technology that enables the drying of blood: the filter paper. Filter paper properties determine the maximum loading capacity, blood spreadability, chromatographic effects, analyte stability and recovery. During the formation of DBS, the content of blood droplets may undergo a chromatographic effect or coffee‐ring effect due to differential diffusion across the filter paper. Besides Hct, other factors such as humidity, drying conditions and material of the filter paper also contribute to the uneven distribution of analytes.^52^ A variety of filter papers with different functionalities are commercially available: (i) standard untreated cellulose papers such as Whatman 903 and Ahlstrom 226, (ii) pre‐treated cellulose papers such as Whatman FTA Elute, FTA DMPK‐A and FTA DMPK‐B for enzyme inhibition or protein denaturation,^53^ (iii) cellulose‐based variants such as water‐soluble carboxymethyl cellulose paper to improve protein precipitation^54^ and (iv) non‐cellulose‐based alternatives such as hydrophilic‐coated woven polyester fibres to provide spot sizes independent of Hct.^55^

Baillargeon et al. developed a patterned DBS (pDBS) card that uses hydrophobic wax barriers to define distinct areas for sample addition, distribution and storage, thereby regulating sample application, distribution and volume control.^56^ These pDBS cards provide an enhanced sampling technology to obtain four uniform replicates independently of the Hct over a broad range of clinical values (20–60%; Figure 2B). Compared to standard DBS cards, the use of pDBS demonstrated a threefold improvement in the accuracy of haemoglobin estimation at low Hct (20%). Additionally, pDBS revealed no statistically significant difference between the recovery of sodium and certain amino acids in dried versus liquid blood samples. A potential downside of their pDBS is the incompatibility of the wax barriers with organic solvents (e.g. methanol, acetonitrile) and harsh surfactants (e.g. Triton X‐100, sodium dodecyl sulphate) which could interfere with accurate sample analysis if leached into the sample during extraction.^56^ A commercially‐available microsampling device that allows for multiple punches from a single sample, similar to the pDBS technology, is HemaSpot^TM^ HD.^57^ HemaSpot^TM^ HD consists of a large membrane which reduces the Hct effect and enables the collection of DBS of ∼160 µL. The greater surface area provides an opportunity to run a variety of assays on a single sample via multiple punches. The membrane is incorporated in a moisture‐tight cartridge with a built‐in desiccant to facilitate a robust storage and shipping solution.^57^

Although different modifications to traditional DBS cards exist to minimise the Hct effects, the easiest approach to eliminate the Hct bias related to spot size and inhomogeneity is to analyse the complete DBS spot formed from a volumetric application of blood. Volumetric DBS can be obtained either by punching the entire DBS after the volumetric application of blood or by volumetrically applying blood on pre‐punched discs.^49^ Accurate volumes of blood for application can be procured using a micropipette or microfluidic channels. However, effective pipetting requires skilled personnel, which limits its scope of application due to the reduced feasibility of self‐sampling. In contrast, microfluidic channels are increasingly being used in the development of self‐sampling‐compliant microsampling devices to produce volumetric DBS for analysis. HemaXis^TM^ DB 10,^58^ Capitainer® B^59^ and HemaPEN®^60^ are commercially‐available devices for volumetric DBS collection that operate on the principle of passive volumetric control using microfluidic channel geometry. Blood obtained from a finger prick by lancet is introduced to the microfluidic channel for initiation of DBS collection. The contents of the filled channels are subsequently transferred onto a standard filter card in HemaXis^TM^ DB 10 and pre‐punched paper discs in Capitainer® B and HemaPEN®. Another device for volumetric DBS collection is the HemaSpot^TM^ HF which is based on the principle of absorbent paper membrane. The fan‐shaped design of the collection matrix with eight blades made up of the HemaForm^TM^ absorbent paper promotes the uniform distribution and volumetric collection of blood droplets applied to its surface.^61^

Although these microsampling devices provide a robust approach for collecting volumetric DBS samples to overcome the Hct spot size and inhomogeneity bias, they fail to eliminate the impact of Hct on the extraction efficiency of analytes. DBS with high Hct tends to create a barrier for extraction, resulting in decreased recovery and an underestimation of analyte concentration.^49^, ^62^ Therefore, optimization of extraction procedures and evaluation of analyte recovery and quantitation accuracy at different Hct levels are absolute necessities during DBS assay validation to ensure that biases are within acceptable limits. Carniel et al. evaluated the influence of Hct at levels 30%, 40% and 50% on the accuracy and recovery of clozapine and norclozapine from DBS samples.^29^ The extraction efficiency was observed to be in the range of 63–67% for clozapine and 58–69% for norclozapine with no significant impact of Hct. The accuracy over all Hct levels was between 98% and 105%, which was within acceptable limits. Other recent studies have also determined that the effect of Hct on DBS sampling is within acceptable limits for a variety of analytes; 8‐isoPGF_2α_, 8‐isoPGE_2_ and PGE_2_ [Hct 30–60%],^22^ tacrolimus [Hct 18–55%],^63^, ^64^ creatinine [Hct 18–55%],^63^ mycophenolic acid [Hct 23–53%],^64^ phenylalanine and tyrosine [Hct 29–64%]^25^ and vancomycin, meropenem and linezolid [Hct 15–40%].^65^ However, research by Chiu et al. on the influence of Hct ranging from 15% to 65% on the quantification accuracy of four monoclonal antibodies (mAb) demonstrated a need for Hct‐based concentration correction to obtain accurate quantification results.^31^ The study exhibited a positive bias exceeding 50% for Hct lower than 25% and a negative bias exceeding 30% for Hct higher than 55%. The bias was attributed to the large size of therapeutic mAbs, which prevented them from passing through the cell membranes of erythrocytes, resulting in their predominant distribution in plasma and an obvious Hct effect. As demonstrated by these different studies, utilization of DBS samples necessitates analyte‐specific validation for the Hct effect. Analyte‐specific validation for Hct effect is particularly important because Hct levels can vary significantly among individuals and even within individuals at different times.^66^ The validation would ensure that laboratory test results are accurate, even in samples with varying Hct levels, allowing clinicians to make informed decisions based on reliable diagnostic information to provide high‐quality patient care. However, analyte‐specific validation requires analysis of samples at various Hct levels, which can be time‐consuming and labour‐intensive. The process would incur additional testing expenses and require specialised equipment for the accurate measurement of Hct.

Despite the inconveniences caused by Hct, several advancements in the DBS technology have been made to improve existing features and develop new functionalities. A new DBS method was recently developed by Ten‐Doménech et al. for accurate, simultaneous quantification of oxidised and reduced glutathione by addressing the challenge of glutathione instability caused by rapid enzymatic or non‐enzymatic oxidation in‐sample.^23^ In this study, a 6‐mm‐diameter filter paper was pre‐treated with 8 µL of N‐ethylmaleimide before DBS sampling to allow immediate derivatization of glutathione at room temperature. This on‐spot derivatization technique prevented the oxidation of glutathione‐to‐glutathione disulphide in samples, circumventing the need for manual intervention by clinical staff for sample derivatization. The method was validated for two clinical scenarios: short‐term storage using residual blood volumes from newborn screening processed after storage at 4°C for 24 h and long‐term storage using cord blood samples processed after storage at −20°C within 1 month. The accuracy and precision of glutathione and glutathione disulphide measurements using this approach were comparable to those of conventional liquid‐blood analyses. However, technological advances are application‐specific and must be validated to ensure comparable outcomes to conventional approaches. For example, Nguyen et al. compared the performance of a smart DBS sampler for on‐paper trypsin digestion to that of the traditional in‐solution trypsin digestion and found the latter to be superior for proteomic profiling.^67^ Although the smart sampler, created by immobilizing trypsin on KIO_4_‐functionalised DBS filter paper, demonstrated that covalently‐bound trypsin had significantly better stability with low autolysis, its performance was discovered to be protein‐dependent. Digestion of a simple protein, such as cytochrome C, produced similar amounts of peptides in both on‐paper digestion and in‐solution digestion, whereas digestion of proteins with disulphide bridges, such as bovine serum albumin, produced more peptides in solution. Due to immediate digestion occurring before reduction and alkylation in the smart sampler, on‐paper digestion displayed lower protein coverage compared to in‐solution digestion.

An alternative to eliminating the Hct effect is the collection of plasma or serum instead of whole blood onto the filter paper. The dried plasma spot (DPS) or dried serum spot (DSS) allows the collection and preservation of the liquid fraction of blood on filter paper without interference from the cellular components.^68^ Similar to DBS, DPS and DSS offer several advantages over traditional plasma or serum samples, including improved stability, ease of collection and transportation, reduced costs and compatibility with automated analysis. However, the conventional DPS and DSS technologies require centrifugation to produce plasma or serum, which can be an expensive and time‐consuming component in any workflow. Alternatively, the collection of plasma or serum from whole blood can be enabled without the requirement for centrifugation by employing passive separation membranes to capture the RBCs while allowing plasma or serum to flow through onto the collection matrix for the formation of DPS or DSS. There are two commercially‐available devices based on this blood separation technology: Telimmune Card and HemaSpot^TM^ SE. The Telimmune card, formerly known as the Noviplex card, comprises a spreading layer and a separation membrane for the removal of blood cells through mechanisms of adsorption and filtration for the volumetric collection of plasma (Figure 2C).^69^ The spiral‐shaped membrane design of HemaSpot^TM^ SE allows for the separation of whole blood into its constituent parts of cells and serum using lateral flow (Figure 2D). Multiple punches for different blood components are feasible at various locations of the membrane; RBCs, platelets and leukocytes in the centre; and serum and components in the spiral arm.^70^ These devices provide higher sample stability and quality by preventing haemolysis and contamination, which can occur during sample collection or transportation of conventional samples.

#### 2.1.2 Dried blood spheroids

**FIGURE 3:**
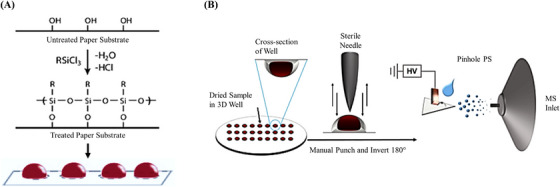
Dried blood spheroid technology. (A) Silanization of hydrophilic cellulose paper to form 3D blood spheroids. (B) Pinhole paper spray mass spectrometry platform using embossed well‐dried blood spheroid. (Adapted from References 73 and 74)

The principal cause of most DBS limitations is the interaction between components of blood and the planar filter paper, which leads to complications in sample quality, analyte extraction and analyte quantification, as discussed previously. In addition, due to the constant exposure of DBS to ambient air, they are highly susceptible to oxidative stress, rendering them unsuitable for the analysis of labile analytes.^71^ These pitfalls of DBS could be overcome with the use of 3D dried blood spheroids, a blood collection technology utilizing hydrophobic paper substrates developed by Damon et al.^72^, ^73^ These hydrophobic substrates are produced by a gas‐phase silanization process on hydrophilic cellulose papers. When blood droplets are applied to these substrates, they bead to form 3D spheroids due to the lower surface energy of the substrate (Figure 3A). As the spheroid begins to dry, evaporation of water causes intact RBCs to self‐assemble onto its surface. These surface RBCs begin to lyse, forming a thin layer of passivation that protects the interior bulk of the sample against environmental stressors, thereby increasing the stability of labile analytes. This technique also eliminates the need for cold storage and laborious sample pre‐treatment by allowing direct sample analysis by PSI‐MS for sensitive analyte quantification with reduced sample volumes.^71^, ^72^

Recent advances in spheroid technology include the development of a spheroid‐based microsampling device by Frey et al. to facilitate volumetric blood collection with short drying durations.^74^ In this study, before the silanization process, the paper substrate was embossed with 3D‐printed templates to create 20 µL wells to collect whole‐blood spheroids. The device included a drying chamber to house the paper substrate containing blood spheroids and 50 µL of ethanol solution for rapid drying facilitated by the increased rate of evaporation of ethanol–water mixtures. Each embossed well incorporating a dried blood spheroid was punched out, inverted onto a second triangular hydrophobic paper and punctured using a sharp 16‐gauge hypodermic sterile needle to provide access to the blood components existing in the semi‐solid state, for pinhole PSI‐MS/MS analysis (Figure 3B). Cocaine and its metabolite benzoylecgonine were used as model chemical systems to evaluate the device because cocaine had been shown to be labile in DBS.^71^ The approach was validated by comparing conventional hydrophilic DBS coupled to PSI‐MS/MS and traditional LC‐ESI‐MS/MS with liquid–liquid extraction. When compared to samples made with planar hydrophilic paper, the sample stability and sensitivity (∼10×) were found to be superior in dried blood spheroids.^74^ Additionally, LC‐ESI‐MS/MS experiments were found to be ∼10× more sensitive with lower LOD and LOQ (cocaine: 0.015 and 1.33 ng/mL; benzoylecgonine: 0.028 and 2.22 ng/mL) compared to pinhole PSI‐MS/MS experiments (cocaine: 0.12 and 2.88 ng/mL; benzoylecgonine: 0.49 and 4.43 ng/mL). The lower sensitivities are primarily attributable to the intrinsic matrix effects resulting from the direct analysis of whole blood in PSI‐MS/MS as compared to the reduced matrix effects resulting from prior sample clean‐up procedures in LC‐ESI‐MS/MS. The clinical efficacy of the spheroid technology was further demonstrated by Sham et al. who measured creatinine levels in whole‐blood spheroid microsamples obtained from adult human volunteers.^21^ The study compared the PSI‐MS/MS analysis of 5 µL of oven‐dried whole blood spheroid collected on a planar hydrophobic paper to the conventional UPLC‐MS/MS analysis of 50 µL of liquid whole blood. Similar results were obtained from both approaches with a maximum deviation of 0.3 µg/mL. The spheroid approach exhibited excellent linearity (*R* ^2^ > 0.99; 2.5–20 µg/mL) across the expected human concentration range, a lower limit of quantification of 2.5 µg/mL, precision ≤ 6.3% and recovery in the range of 88–94%.^21^

#### 2.1.3 Volumetric tip microsampling

**FIGURE 4:**
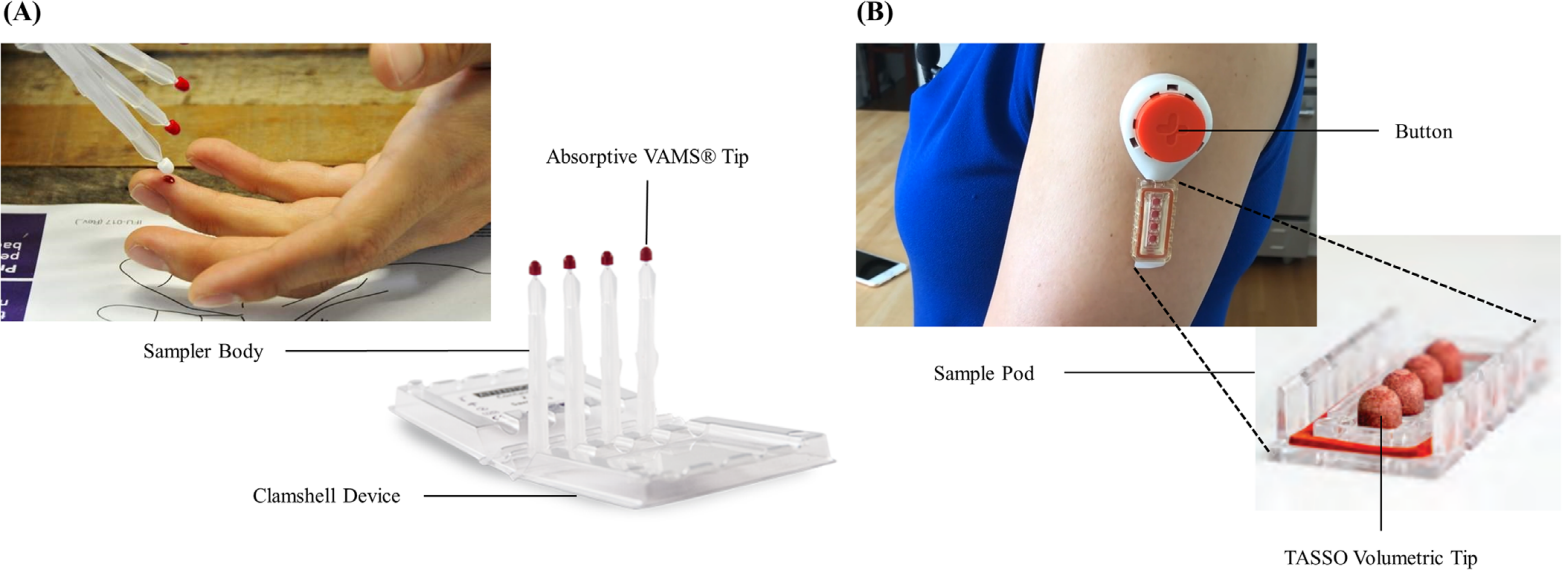
Volumetric tip microsampling technology. (A) Mitra device by Neoteryx. (B) TASSO‐M20 device by TASSO Inc. (Adapted from References 75 and 76)

Volumetric tip microsampling (VTM), introduced to the market in 2014, is an alternative microsampling technique to collect Hct‐independent, fixed‐volume dried whole‐blood samples by accurate and precise absorption of blood into a volumetric polymer tip. The volume of blood collected is controlled by the amount and properties of the polymeric substrate. After the samples collected on these tips are dried, they are stored or transported to the laboratory for analysis.^14^ The VTM technique is currently employed by two commercially‐available blood collection devices: Mitra and TASSO‐M20. The Mitra device comprises a plastic sampler attached to a proprietary hydrophilic polymer tip which functions based on their patented volumetric absorptive microsampling (VAMS®) technology. To initiate sample collection, the leading surface of the Mitra tip is dipped into blood obtained via a finger prick (Figure 4A).^75^ The Mitra devices are compatible with 96‐Autoracks and automated liquid handling systems which facilitate high‐throughput testing. The TASSO device consists of a large button, a lancet, microfluid channels and a sample‐collection pod with four TASSO volumetric tips. For TASSO‐M20 blood collection, the device adheres to the upper arm of the patient and the button is pressed firmly and released, which creates a vacuum and deploys the lancet to puncture the arm to draw blood which is directed to the tips via the capillary channels (Figure 4B).^76^ With the use of VTM for sample collection, issues of inhomogeneity experienced by DBS sampling are eliminated, as there is no filter paper component to cause the biases, such as uneven spreading, distribution and sample size variability.

However, certain aspects of the VTM technology require careful consideration prior to its implementation. A critical and comprehensive overview of the practical aspects of VAMS can be found in the 2018 review by Kok et al.^77^ One of the potential challenges of VTM is the difficulty in the extraction of analytes which could be trapped in the crevices of the porous polymer tip due to the possibility of dried erythrocyte occlusion.^25^ However, studies have shown that additional processes such as pre‐wetting the polymer tip with water and sonication during sample preparation can improve the extraction efficiency of analytes.^77^, ^78^ Another limitation with VTM is the inability to add stabilizing agents during sample collection for the accurate quantification of unstable analytes susceptible to hydrolytic degradation. Addition of reagents to the polymer tips prior to sample collection could potentially result in undesirable sample volumes and inhomogeneous distribution of analytes. Nevertheless, different drying conditions, such as the use of a desiccator, pressurization, nitrogen gas and household vacuum sealer, have been shown to improve sample stability. In the analysis of acetylsalicylic acid, a hydrolysis‐prone analyte, Moon et al. showed that humidity control was critical for its stability, and the use of a simple household vacuum sealer could maintain the stability for up to 1 month.^33^

Similar to DBS filter paper modifications, the polymeric tips can be modified to attain new features. Comparable to the smart DBS sampler for proteomics, Reubsaet et al. modified the Mitra device to build a smart VAMS proteolysis sampler. Maximum proteolytic sampler activity was achieved when at least 50 µg of trypsin was covalently bound to the Mitra tip at a pH of 9. The study observed no significant difference in the absorbed sample volume or drying rate even though the tips were subjected to chemical modifications before sampling. A comparison of in‐solution trypsin digestion with the VAMS digestion for seven proteins revealed higher intensities of tryptic peptides in VAMS digestion for four proteins. The smart sampler was also applied to complex serum samples with overnight digestion, which yielded similar peptide and protein identifications to in‐solution digestion, demonstrating the potential of modified VAMS for proteomics sample preparation.^79^

### 2.2 Liquid matrix microsampling

An overview of commercially‐available liquid matrix microsampling devices can be found in Table 2.

**TABLE 2:** Overview of commercially‐available liquid matrix microsampling devices.

Technology	Device	Company	Design	Volume	Samples	Advantage
Capillary‐based	Minivette POCT	Sarstedt AG & Co. KG (Numbrecht, Germany)	Plastic volumetric capillaries	10–200 µL	1	Collection of volumetric liquid whole blood; compatible for volumetric DBS collection
	Microvette APT	Sarstedt AG & Co. KG (Numbrecht, Germany)	Plastic capillary or a collection rim	250–500 µL	Multiple	Tubes compatible with automated sample processing in blood count analysis systems
	Microsampling Wing	Shimadzu Europa (Duisburg, Germany)	Olefin polymer U‐shaped capillary microchannel with H‐shaped segment at the top with an inlet port	23 µL whole blood (5.6 µL plasma (2.8 µL + 2.8 µL))	1 or 2	Easy plasma fractionation; low human error
Tube‐based	TASSO+	Tasso Inc. (Seattle, United States)	Device with button, lancet, microfluidic channels connected to standard collection tubes	250–600 µL	Multiple	Compatible with multiple standard collection tubes
	TASSO‐SST	Tasso Inc. (Seattle, United States)	Device with button, lancet, microfluidic channels connected to tube with serum separator gel with no anticoagulants	200–300 µL	Multiple	Collection of serum
	TAP II	YourBio Health, Inc. (Medford, United States)	Device with button, solid microneedle array, connected to standard collection tubes	300–900 µL	Multiple	Compatible with multiple standard collection tubes; skin puncture via solid microneedles‐less painful

#### 2.2.1 Capillary microsampling

**FIGURE 5:**
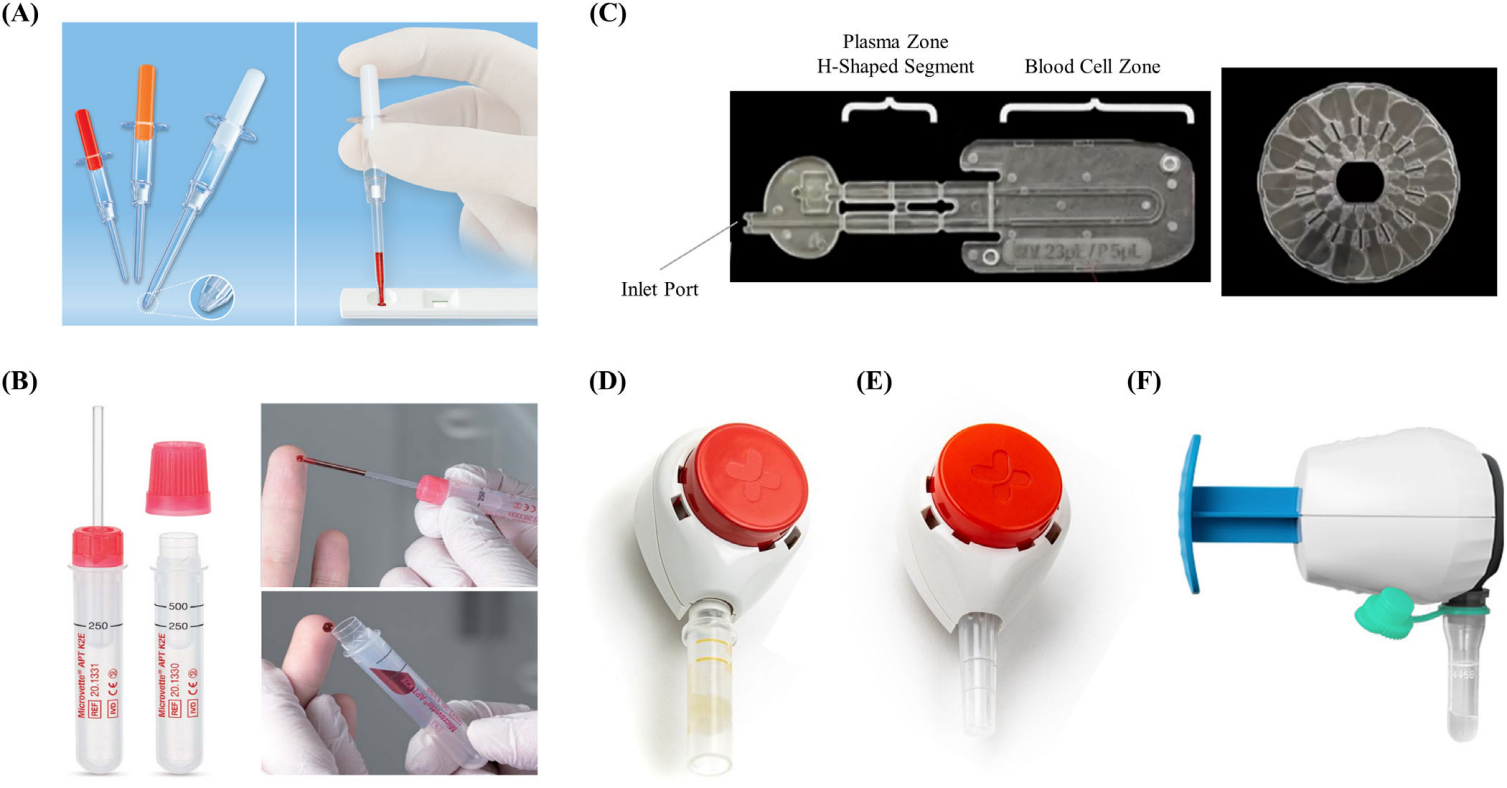
Liquid matrix microsampling technology. (A) Minivette® POCT. (B) Microvette® APT. (C) Microsampling wing and microsampling windmill. (D) TASSO+. (E) TASSO‐SST. (F) TAP II. (Adapted from References 82–87)

Capillary microsampling (CMS) is a versatile and easily implemented technique for the collection of small, accurate volumes of liquid matrices, including whole blood.^80^ The CMS technology enables the collection of accurate blood volume using exact‐volume capillaries, usually coated with anticoagulants, to take up blood obtained via finger prick through capillary forces. The filled capillaries can be stored in sample tubes for later use or prepared for immediate use. One of the commonly employed pre‐treatment procedures is centrifugation for the generation of plasma. Typically, the capillaries are sealed with wax to prevent sample loss during centrifugation. After centrifugation, the plasma samples can be transferred using a micropipette into a sample tube or an end‐to‐end transfer into another capillary.^81^ CMS is compatible with well‐established sample‐preparation techniques, including the addition of agents for immediate stabilization of unstable compounds, and laboratory automation.^80^ Some of the commercially‐available CMS devices are the Sarstedt Tubes, such as the Minivette® POCT series and Microvette® APT, and the Microsampling Wing (MSW). The Minivette® POCT series consists of plastic capillaries which are available in a range of sample volumes (10 µL–200 µL) and anticoagulants for preparation to facilitate the simple collection and dispensing of blood for point‐of‐care tests (Figure 5A).^82^ The Microvette® APT enables the collection of K_2_ EDTA whole blood (250–500 µL) directly into the tube for routine analysis either via end‐to‐end capillary filling or a collection rim. It is specially designed to facilitate automated sample processing in blood count analysis systems (Figure 5B).^83^ The MSW device comprises a U‐shaped capillary microchannel with an H‐shaped segment at the top containing EDTA anticoagulant for the volumetric collection of whole‐blood samples which can be introduced through an inlet port. Upon centrifugation of the device, the plasma is retained in the H‐shaped segment which can be snapped into two separate segments to provide two volumetrically accurate plasma samples. Additionally, a specialised holder called the Microsampling Windmill allows for the simultaneous and efficient centrifugation of 14 MSWs at a time (Figure 5C).^84^

#### 2.2.2 Tube microsampling

Tube microsampling (TMS) involves the collection of liquid blood microsamples directly into microtubes. Three commercially‐available devices use the TMS technology: TASSO+, TASSO‐SST and TAP II devices (Figure 5D–F). The design and operation of these TMS‐based devices are similar to the TASSO‐M20 device. Instead of the sample pod, the TASSO+ and TASSO‐SST devices consist of appropriate microtubes with or without anticoagulants to facilitate the downstream collection of plasma (TASSO+) or serum (TASSO‐SST).^85^, ^86^ These devices are compatible with a variety of additive microtubes. The difference between the TAP II device and the TMS‐based TASSO devices is the skin‐puncturing mechanism during the blood collection procedure. Instead of a button‐activated lancet deployment, the TAP II device deploys a solid microneedle array for skin puncture and a vacuum for blood flow into the microtubes.^87^ Microneedles are known to be less painful than lancets for the skin puncture due to their micron‐sized dimensions which prevent them from hitting the nerve endings when inserted into the skin.^88^ These single‐use, button‐activated devices enable at‐home self‐sampling of up to 500 µL of blood into microtubes which can be shipped to the laboratory for further analysis. Larger volumes of blood may be collected by these devices in the future if their designs are modified to create stronger vacuum or larger negative pressure.

## 3 APPLICATIONS

The following section summarises microsampling research published in the year 2022 in the field of volume‐limited applications, TDM, biomarker research, forensic toxicology and sports anti‐doping. An overview of the research articles on method development and validation studies can be found in Table 3.

**TABLE 3:** Overview of microsampling research in biomedical applications published in the year 2022.

Application	Technology	Device	Sample volume	Target compound	Analytical method	Reference
**Animal studies**	DBS using CMS	Glass capillary and Whatman 903	∼	Clozapine	LC‐MS	[94]
	VTM	Mitra	10 µL	Vincristine and tariquidar	LC‐MS/MS	[27]
	CMS	Heparinised capillary tubes	10 µL	E7130	UHPLC‐HRMS	[93]
	CMS	Glass microcapillaries	15 µL	Trans‐resveratrol and its main metabolites	HPLC‐MS/MS	[92]
**Neonatal and paediatric applications**	∼	∼	10 µL	Thiotepa, tepa, cyclophosphamide (CP), and 4‐hydroxycyclophosphamide (4‐OHCP)	LC‐MS/MS	[102]
	DBS	Whatman 903	8 mm punch (∼17 µL)	Gentamicin sulfate	UHPLC‐MS/MS.	[100]
	DBS	Whatman 903, Minivette POCT, HemaXis, Capitainer	6 mm punch, 20 and 50 µL, 10 µL, 13.5 yL	Isoprostanoids 8‐iso prostaglandin F2α and 8‐iso prostaglandin E2, and prostaglandin E2	UHPLC‐MS/MS	[22]
	DBS	∼	10 µL	Oxidised and reduced glutathione	LC‐MS	[23]
	DBS	Perkin Elmer 226, HemaXis, Capitainer, Mitra	3.2 mm punch and varying volumes tested	Phenylalanine and tyrosine	FIA‐MS/MS	[25]
	VTM	Mitra	10 µL	Gentamicin	LC‐MS/MS	[101]
	VTM	Mitra	10 µL	Furosemide	UHPLC‐MS/MS	[103]
	CMS	Microsampling Wing and Li‐H capillary tube	5.6 µL	Meropenem	LC‐MS/MS	[30]
	DBS and CMS	Capillary and Whatman 903	50 µL and 10 mm punch	Vancomycin, meropenem and linezolid	UHPLC‐MS/MS	[65]
	CMS	EDTA coated capillary	70 µL	Tafenoquine	LC‐MS/MS	[105]
	CMS	heparinised plastic capillary tubes	50 µL	Cefotaxime and desacetylcefotaxime	UHPLC‐MS/MS	[26]
**Therapeutic drug monitoring**	VTM	TASSO‐M20	20 µL	Tacrolimus and mycophenolic acid	LC‐MS/MS	[113]
	DBS and VTM	Whatman 903 and Mitra	3 mm punch and 20 µL	Tacrolimus and creatinine	LC‐MS/MS	[63]
	DBS and VTM	HemaXis and Mitra	10 µL	Clozapine	HPLC‐ED	[126]
	VTM	Mitra	∼	Hydrochlorothiazide, furosemide, lisinopril, and torsemide, as well as the metabolites canrenone, enalaprilat, and ramiprilat	LC‐MS	[127]
	VTM	Mitra	20 µL	Methotrexate and methotrexate polyglutamates [MTX‐PG_1‐5_ and MTX‐PG_6‐7_]	LC‐MS/MS	[28]
	DBS and DPS	HemaXis and Whatman 903	10 µL and 6 mm punch	clozapine and norclozapine	HPLC‐MS/MS	[29]
	VTM	Mitra	30 µL	Cannabidiol (CBD), tetrahydrocannabinol (THC) and their respective metabolites: cannabidiol‐7‐oic acid (7‐COOH‐CBD); 7‐hydroxy‐cannabidiol (7‐OH‐CBD); 6‐alpha‐hydroxy‐cannabidiol (6‐α‐OH‐CBD); and 6‐beta‐hydroxycannabidiol (6‐β‐OH‐CBD); 11‐ Hydroxy‐Δ9‐tetrahydrocannabinol (11‐OH‐THC) and 11Nor‐9‐carboxy‐Δ9‐tetrahydrocannabinol (THCCOOH)	UHPLC‐MS/MS	[131]
	DPS	∼	∼	methotrexate	LC‐MS/MS	[117]
	DBS and VTM	Whatman 903 and DMPK‐C, HemaXis, Capitainer‐B, and Mitra	∼	Tacrolimus, ciclosporin, everolimus, sirolimus and mycophenolic acid	LC‐MS/MS	[115]
	DBS	Whatman 903	15 µL	Bevacizumab, trastuzumab, nivolumab and tocilizumab	LC‐MS/MS	[31]
	DBS using CMS	Capillary micropipette and Whatman 903	10 µL	Tacrolimus, sirolimus, everolimus and cyclosporin A	LC‐MS/MS	[112]
	TMS	TASSO‐SST	∼	CRP, RF IgM, CCP IgG	Phadia^TM^ 250 instrument and Thermo Scientific™ Indiko™ Plus Clinical Chemistry Analyzer	[24]
	DBS and VTM	Whatman 903 and Mitra	6 mm punch and 20 µL	Tacrolimus and mycophenolic acid	LC‐MS/MS	[64]
	VTM	Mitra	20 µL	Imatinib mesylate	LC‐MS/MS	[125]
	VTM	Mitra	10 µL	Capecitabine, 5′‐deoxy‐5‐fluorocytidine, 5′‐deoxy‐5fluorouridine and 5‐fluorouracil	LC‐MS/MS	[40]
	VTM	Mitra	10 µL	Bosutinib, dasatinib, gilteritinib, ibrutinib, imatinib, midostaurin, nilotinib and ponatinib	LC‐MS/MS	[78]
	VTM	Mitra	20 µL	Axitinib	LC‐MS/MS	[120]
	VTM	Mitra	20 µL	Cabozantinib, dabrafenib, nilotinib, osimertinib, afatinib, axitinib, bosutinib, lenvatinib, ruxolitinib and trametinib	LC‐MS/MS	[121]
	VTM	Mitra	∼	Acetylsalicylic acid	LC‐MS/MS	[33]
	DBS and VTM	HemaXis and Mitra	10 µL and 20 µL	Tacrolimus, mycophenolic acid, creatinine and iohexol	LC‐MS/MS	[32]
	VTM	Mitra	10 µL	Paracetamol and its four metabolites: paracetamol‐glucuronide (APAP‐Gluc), paracetamol‐sulphate (APAP‐Sulf), paracetamol‐mercapturate (APAP‐Merc), and paracetamol‐cysteine (APAP‐Cys)	LC‐MS/MS	[128]
	DPS	Telimmune Card	∼70 µL	Giredestrant	LC‐MS/MS	[118]
	VTM	Mitra	∼	Favipiravir	HPLC‐Diode Array Detector	[129]
	VTM	Mitra	30 µL	Cyclophosphamide (CP), and 4‐hydroxycyclophosphamide (4‐OHCP)	UPLC–MS/MS	[119]
**Omics‐based biomarker research**	VTM	Mitra	20 µL	Mercury	DMA80‐evo® Analyzer	[41]
	DBS	Whatman 903	30 µL and 50 µL	Mercury	Thermal Decomposition Amalgamation and Atomic Absorption Spectrometry (TDA‐AAS) in a Direct Mercury 18 Analyzer (DMA)	[145]
	VTM	Mitra	30 µL	Antibodies against SARS‐CoV‐2 nucleocapsid (IgG), spike protein (IgG, IgM, IgA), and receptor binding domain (IgG, IgM, IgA)	enzyme linked immunosorbent assay	[144]
	DBS and VTM	HemaXis and Mitra	10 µL	SARSCoV‐2 anti‐Spike IgG antibody	microfluidic nano‐immunoassay	[143]
	TMS	TASSO‐SST	∼	17 protein inflammatory biomarkers including CRP, Ferritin, IL‐6, PCT, D‐dimer, IL‐1B and IL‐1Ra	Ella multi‐analyte immunoassay	[42]
	DBS and VTM	Whatman 903 and Mitra	20 µL	aflatoxin B1	LC‐MS/MS	[141]
	VTM	Mitra	10 µL	proteomic profile	LC‐MS	[134]
	DBS and DPS	Whatman 903 and Telimmune card	6 mm punch and 3.2 µL	498 compounds covering 24 lipid subclasses	UHPLC‐MS/MS	[132]
	VTM	Mitra	∼	1600 proteins	LC‐MS	[135]
	VTM	Mitra	10 µL	24 compounds related to tryptophan metabolism	LC‐MS/MS	[138]
	TMS	TASSO‐SST	∼	Uracil	LC‐MS/MS	[137]
	VTM	Mitra	30 µL	Acrylamide and glycidamide	HPLC‐UV	[140]
	VTM	Mitra	∼	Testosterone, cortisol, 25 hydroxyvitamin D and bone resorption marker β‐CTX	LC‐MS/MS and COBAS 6000 e601 using electro‐chemiluminescence immunoassay	[139]
	VTM	Mitra	10 µL	35 metabolites including taurine, serotonin, hypoxanthine, glutathione, aurine, 1‐methyl histidine, acetyl‐carnitine, and hypoxanthine	LC‐MS	[136]
	Dried Blood Spheroid	∼	5 µL	Creatinine	PSI‐MS/MS	[21]
**Forensic toxicology**	DBS and VTM	Whatman 903 and Mitra	20 µL	Continuous erythropoietin receptor activator (CERA)	ELISA kit	[38]
	Dried Blood Spheroid	∼	20 µL	Cocaine and its metabolite benzoylecgonine	PSI‐MS/MS	[74]
	DBS	Whatman FTA^TM^	20 µL	425 drugs including including benzodiazepines, antipsychotics, antidepressants, antipyretic analgesics, non‐steroidal anti‐inflammatory drugs, antibiotics, antiepileptic drugs, and new psychoactive drugs	LC‐MS/MS	[153]
	DBS and VTM	Whatman 903 and TASSO‐M20	60 µL	Endogenous erythropoietin (hEPO) and prohibited ERAs (BRP, NESP, CERA and EPO‐Fc)	SAR‐PAGE and Western blotting	[37]
	DBS	∼	30 µL	PEth	UHPLC‐QTOF‐HRMS and UHPLC‐MS/MS	[148]
	DBS and VTM	HemaXis and Mitra	∼	Peth 16:0/18:1, PEth16:0/20:4 and ethylglucuronide (EtG)	LC‐MS/MS	[150]
	DBS	∼	∼	13 anabolic steroid esters	DBSA‐TLX‐HRMS	[158]
	DBS using CMS	Whatman 903 and capillary	20 µL	PEth‐homologues (16:0/18:1; 16:0/18:2; 16:0/20:4; 17:0/18:1; 18:0/18:1; 18:1/18:1; 18:0/18:2)	LC‐MS/MS	[151]
	DBS	HemaXis	10 µL	PEth 16:0/18:1	LC‐HRMS	[149]
	DBS	Whatman	30 µL	ricinine and L‐abrine	LC‐MS/MS	[155]
	DBS and TMS	∼	∼	Aluminium (Al), total arsenic (As), cadmium (Cd), cobalt (Co), chromium (Cr), copper (Cu), manganese (Mn), molybdenum (Mo), nickel (Ni), lead (Pb), selenium (Se) and zinc (Zn)	ICP‐MS	[156]
	DBS	FTA™ DMPK C	30 µL	132 synthetic opioids, cathinones, hallucinogens & fentanyl	UHPLC‐HRMS‐QTOF	[34]
	DBS and VTM	HemaSpot‐HF, Whatman 903 Protein Saver cards, Whatman FTA DMPK‐A, B and C cards, Tasso‐M20, and Mitra	Varying volumes	235 drugs	UHPLC− HRMS	[39]
	DBS	HemaXis	10 µL	PEth 16:0/18:1	LC‐MS/MS	[35]
	DBS	HemaPEN	2.74 µL	Cocaine and the metabolites benzoylecgonine and cocaethylene	LC‐MS/MS	[152]
	VTM	Mitra	20 µL	85 licit and illicit drugs	LC‐HRMS	[154]

CCP, cyclic citrullinated peptide; CMS, capillary microsampling; CRP, C‐reactive protein; DBS, dried blood spot; DPS, dried plasma spot; ED, electrochemical detection; FIA, flow injection analysis; HRMS, high‐resolution MS; ICP, inductively coupled plasma; Ig, immunoglobulin; IL‐1β, interleukin‐1 beta; IL‐Ra, interleukin‐1 receptor antagonist; Li‐H, lithium‐heparin; PCT, procalcitonin; PSI, paper spray ionisation; RF, rheumatoid factor; UHPLC, ultra‐high performance LC; VTM, volumetric tip microsampling; ‘∼’, not available.

### 3.1 Volume‐limited applications

#### 3.1.1 Animal studies

Small animals are widely employed in pre‐clinical research for drug development for PK, PD, toxicokinetic (TK) and toxicodynamic (TD) investigations to determine the bioavailability of drugs at pharmacological concentrations and ensure drug safety. These studies typically require collection of blood samples from a single animal candidate at several timepoints, which can lead to health deterioration or even early death, due to extensive loss of blood. To protect the well‐being of animals used in research, the Universities Federation for Animal Research advocated the 3Rs principle: Replace, Reduce and Refine.^89^ Making animal studies more ethical through the 3Rs can be achieved with microsampling, as it reduces stress, sampling time and the number of animals needed for studies.^90^ Guidelines that consolidate the regulatory perspectives on microsampling and support its use in blood sampling have also been added to and reviewed by the International Council for Harmonization of Technical Requirements for Pharmaceuticals for Human Use.^91^

Different microsampling technologies have been employed in recent investigations by Rosser et al., Xu et al., Ryu et al. and Pelcovà et al. in animal studies for PK applications. In the work of Rosser et al., the PK assessment of vincristine and tariquidar in mice using an LC‐MS/MS approach was effectively carried out using the VAMS technology. Assessment of both single‐agent therapy and combination therapy over a 24 h period in 10 µL microsamples revealed a 2.3‐fold increase in vincristine drug exposure when combined with tariquidar, which validated the use of the approach for longitudinal analysis of drug exposure in animal studies.^27^ Xu et al. evaluated the feasibility of CMS coupled to HPLC‐MS/MS for mice PK studies using trans‐resveratrol as the model drug. The PK parameters of the drug and its associated metabolites found in the study were comparable with those reported in previous studies, which demonstrated that CMS provides credible samples for subsequent quantitative PK analysis.^92^ Similarly, Ryu et al. also successfully employed CMS in the PK study of E7130 in mice using an HRMS quantification method.^93^ Additionally, a local and national animal welfare committee approved PK pilot study of clozapine was successfully conducted on a single albino rat using DBS samples spotted using CMS.^94^

Traditionally, satellite animals are used in TK analysis to avoid the effects of blood sampling on toxicological endpoints in main study animals, which leads to a higher number of required study animals. Microsampling has been shown to facilitate toxicity assessment and TK analysis in the same animals, even for hematotoxic compounds. Implementation of microsampling in TK studies is often contested by regulatory bodies due to the unidentified effects of microsampling on TK parameters, necessitating bridging studies.^95^ Tochitani et al. conducted a 2‐week rat study to examine the effects of microsampling on toxicity assessment using methylene blue trihydrate and azathioprine as test compounds. Using urinalysis, haematology, blood chemistry, organ weights and histopathology, the study compared the results of toxicological end points between non‐microsampling and microsampling subgroups. The differences between the subgroups were found to be small and insignificant, indicating that toxicity of hematotoxic compounds could be detected even with microsampling without any overestimation of toxicity.^96^ In another study, during the examination of 109 carcinogenicity studies undertaken for new drug applications involving a minimum of 65,341 mice and rats, Manuppello et al. determined that an 18.7% reduction in animal size could have been accomplished by evaluating exposure by microsampling blood in main study animals as opposed to TK satellites.^97^

#### 3.1.2 Neonatal and paediatric studies

Research in neonatal and paediatric populations is challenging due to their small volume of circulating blood and the physiological and psychological burden associated with blood sampling. Pain and stress in the early phase of life are known to affect the neurodevelopment leading to poor cognitive outcomes and long‐term effects on social–emotional behaviour.^98^ Blood sampling with automatic lancets has been demonstrated to cause less pain in neonates than with needles.^99^ Furthermore, the physiological and pharmacological characteristics of these populations, in particular the preterm infants, differ significantly from adults, which often necessitate tailored dosing regimens for medication administration. However, standard blood sampling for PK studies and TDM may increase the risk of iatrogenic anaemia in critically ill neonatal and paediatric populations. Microsampling may overcome both the ethical and physiological concerns, improving the feasibility of PK studies in these populations to supersede the use of off‐label drugs and dosing regimens extrapolated from clinical studies conducted on adults.

Several assays for the quantification of drugs in microsamples have been developed for use in neonatal and paediatric PK studies. A ultra‐high‐performance LC (UHPLC) MS/MS method capable of identifying distinct gentamicin forms was recently developed by dos Santos et al., allowing for the measurement of their concentrations ranging from 0.1–40 mg/L in non‐volumetric DBS. The stability of gentamicin was determined to be 21 days at −20 and 8°C and 24 h at room temperature. The assay was evaluated with nine neonatal patients which revealed that the difference between the estimated plasma concentration from DBS, determined using the Hct level and a correction factor, and concentration measured using conventional venous plasma was statistically insignificant but highly variable.^100^ Similarly, Parker et al. reported for the first time the use of VAMS in neonatal TDM of gentamicin. The study including two neonates demonstrated that the comparison of calculated gentamicin concentration from VAMS to observed plasma concentration met the acceptance criteria of an incurred sample reanalysis test (≤20%) and results were obtained promptly.^101^ Other LC‐MS/MS assays that have been recently developed include assays for the quantification of thiotepa, its metabolite tepa, CP and its metabolite 4OHCP in 10 µL liquid plasma samples and furosemide in 10 µL VAMS samples.^102^, ^103^

As a part of clinical bridging studies, several studies have compared two or more microsampling approaches for a given neonatal or paediatric application. For the analysis of meropenem, a capillary with lithium heparin anticoagulant was found to perform better than the MSW, although both yielded acceptable concentrations comparable to those obtained from conventional arterial catheter sampling.^30^ In another study, Xiaoyong et al. compared DBS and CMS for the measurement of vancomycin, meropenem and linezolid. The results showed a significant correlation between DBS and CMS measurements (*R* > 0.98; *p* < 0.01) for all three antibiotics. However, CMS to DBS drug concentration ratios were highly variable for vancomycin (∼1.39) and meropenem (∼1.34) which required an Hct correction factor for accurate measurements. The study suggests DBS for unstable, hydrophobic compounds, such as meropenem and linezolid, but CMS for vancomycin due to its low adsorption capability and high stability.^65^ Biagini et al. evaluated non‐volumetric DBS and volumetric DBS (HemaXis^TM^ DB, Minivette POCT, Capitainer® B) for the determination of isoprostanoids and prostanoids in preterm newborns suffering from Patent Ductus Arteriosus. It was found that the Hct biases affected the non‐volumetric DBS measurements. All analytes measured with Minivette POCT exhibited a recovery of 78–108% and a precision of less than 15%, compared to a recovery and precision of 23–81% and 20–25%, respectively, for other volumetric DBS. Based on the analytical performance parameters and cost, the Minivette POCT was found to be the best option for DBS sampling for the application.^22^ Similarly, volumetric blood collection devices (Capitainer® B, HemaXis^TM^ DB 10 and Mitra) were also evaluated for the monitoring of phenylalanine and tyrosine in patients with phenylketonuria in a study by Carling et al. which revealed their superior analytical performance (mean biases in relation to conventional liquid samples: −7.8 (Capitainer® B), −5.1 (HemaXis^TM^ DB 10), −12 (Mitra)) compared to traditional DBS (−32.6).^25^

Microsampling can also facilitate data‐rich modelling and simulations for population PK research.^104^ In a study by Bachhav et al., the population PK models obtained from CMS and venous samples were found to provide the same dosing regimen recommendations for tafenoquine antimalarial in paediatric patients.^105^ Guerra Valero et al. also evaluated the suitability of CMS to describe the population PK of cefotaxime and its metabolite desacetylcefotaxime in critically ill paediatric patients.^26^ The authors further showed that parents and bedside nurses preferred microsampling over conventional sampling for paediatric PK clinical investigations using a seven‐point Likert‐scale questionnaire.^106^ In the field of population‐based research, the use of archival newborn screening DBS samples has shown immense potential for emerging targeted and untargeted multi‐omics analysis.^107^ Microsampling with point‐of‐care testing has also been identified as a blood conservation strategy that significantly reduces the need for adult RBC transfusion therapy for anaemia of prematurity.^108^

### 3.2 Therapeutic drug monitoring

TDM specialises in the measurement of drug concentrations in biological fluids, such as serum, plasma or blood, to facilitate personalised medicine. Individualised drug therapy for improved patient outcomes is ensured through the monitoring of drug concentration profiles in patients to adapt drug dosage, interval and duration to account for potential inter‐individual genetic and metabolic differences in drug responsiveness and the drug concentration‐effect correlation.^109^ Thus, TDM implementation requires timely, rapid and accurate measurements of target drug concentrations to evaluate the clinical therapeutic efficacy and prevent failure (too low concentration) and toxicity (too high concentration). Microsampling has been increasingly employed in TDM to facilitate at‐home, convenient, pain‐free sample collection with easy storage and transport.^110^ Despite being a relatively robust method for routine TDM, implementation of microsampling requires development and validation of bioanalytical assays to obtain good analyte sensitivity, stability, extraction efficiency and correct clinical interpretation of results. Routine TDM is prescribed for a variety of therapeutic drug categories such as immunosuppressants (IMS), antibiotics, anticancer drugs, psychoactive drugs, antidepressants and analgesics.^111^

Microsampling has been extensively studied for the determination of tacrolimus and mycophenolic acid, two of the most commonly prescribed IMS used to prevent organ rejection in recipients of solid organ transplants.^32^, ^63^, ^64^, ^112^, ^113^ Studies conducted by Paniagua‐González et al. and Zwart et al. in liver or renal transplant patients, respectively, found that although DBS and VAMS, after a concentration correction, provided comparable results to conventional whole‐blood tacrolimus and plasma mycophenolic acid measurements, DBS performed better than VAMS in terms of clinical performance (98.7% of paired samples within ±20% difference as opposed to 71.1% of VAMS paired samples), sample quality (1 sample discarded as opposed to 9 VAMS samples due to poor sample quality) and costs (7 times lower than Mitra device).^32^, ^64^ Additionally, use of non‐contact Hct prediction strategies such as NIR and UV/VIS spectroscopy coupled to an automated DBS workflow was found to adequately correct for Hct effect in determining IMS concentrations.^114^ In contrast, for the simultaneous monitoring of tacrolimus and kidney functioning using creatinine, VAMS was found to be the preferred single sampling option compared to DBS, as only 69.4% of paired samples with DBS were within ±15% as opposed to 81.6% with VAMS.^63^ Additionally, Leino et al. also validated the application of TASSO‐M20 for the quantitative bioanalysis of tacrolimus and mycophenolic acid.^113^ Although these studies demonstrated the feasibility of microsampling in TDM of IMS, results from a proficiency testing pilot for IMS microsampling assays when compared to whole blood methods showed great interlaboratory variation. Since the results influence clinical decision‐making, harmonization and standardization are thus necessary before microsampling techniques can be implemented in patient care.^115^ A recent review by Deprez et al. summarises the state‐of‐the‐art for DBS‐based TDM of IMS, including a critical evaluation of challenges, implementation status in clinical practice and considerations to overcome implementation barriers.^116^

One of the clinically relevant characteristics of drugs is their differential distribution between cells (erythrocytes and leukocytes) and plasma, which necessitates the selection of an appropriate matrix for TDM analysis. In the treatment of rheumatoid arthritis, the site of action of methotrexate is assumed to be in the peripheral blood mononuclear cells. A highly sensitive and specific LC‐MS/MS method was developed for the quantification of methotrexate polyglutamates which are active metabolites of methotrexate. Evaluation of methotrexate polyglutamates in different matrices including plasma, RBCs, peripheral blood mononuclear cells and venous whole blood revealed that VAMS can be used for accurate TDM analysis only if sampling is done immediately before the next dose of methotrexate to ensure that concentrations are not overestimated.^28^ In another study in patients with lymphoblastic leukaemia, DPS and wet plasma concentrations of methotrexate were found to have no statistically significant difference.^117^ However, DPS from Telimmune cards have been shown to produce inconsistent filtration across different cards, in the study by Tang et al., for the quantification of giredestrant, stipulating the need for further optimization to ensure good accuracy and precision.^118^ Various LC‐MS/MS assays for the VAMS technology have been developed and validated for the quantification of other chemotherapy drugs and its metabolites such as 5‐fluorouracil, capecitabine, CP, 4‐OHCP and several tyrosine kinase inhibitors (TKIs).^40^, ^78^, ^119^, ^120^, ^121^

Most available TKIs are susceptible to drug–drug interactions, and their bioavailability is dependent on drug formulation, gastrointestinal absorption and concomitant food intake;^122^, ^123^ therefore, the implementation of TDM of TKIs in the standard care of oncology patients has the potential to improve treatment outcomes and reduce toxicity by monitoring medication adherence and actual drug concentration in plasma. A recent review by Verougstraete et al. provides a comprehensive description of the different methodological aspects to consider in dried blood microsample methods for TKI TDM.^124^ The same authors developed an LC‐MS/MS assay for the simultaneous quantification of eight TKIs following substantial optimization to obtain an Hct‐ and storage‐ independent sample preparation procedure.^78^ Opitz et al. utilised an on‐line SPE system to ease the extraction of axitinib from VAMS samples.^120^ Due to a high blood‐to‐plasma ratio of TKIs, conversion factors are necessary to compare VAMS TKI concentrations with conventional plasma concentrations. Zimmerman et al. determined 10 TKIs, where nine TKIs exhibited a constant conversion factor over their entire calibration range with only trametinib exhibiting a decreasing conversion factor with increasing VAMS concentration.^121^ Further, the degree of patient satisfaction concerning self‐collection and treatment adherence using VAMS has been evaluated on chronic myeloid leukaemia patients receiving imatinib mesylate therapy.^125^ The study revealed that 87% of patients experienced less discomfort with VAMS sampling than with conventional sampling but only 77% of patient‐collected samples met the quality criteria.

In their respective studies, Carniel et al. and Marasca et al. examined DBS, DPS and VAMS technologies for the determination of antipsychotic drugs, clozapine and norclozapine. DBS and DPS measurements were found to be highly correlated to serum levels, with greater accuracy for DPS compared to DBS.^29^ A 30‐day stability test showed higher stabilities in the dried matrices stored at room temperature when compared to liquid plasma samples stored under the controlled temperature of −20°C.^126^ Further, the VAMS‐based strategy was shown to be suitable for assessing adherence to several classes of antihypertensive drugs and quantification of paracetamol and favipiravir.^127^, ^128^, ^129^ Microsampling has also been applied for the determination of tetrahydrocannabinol, cannabidiol and their respective metabolites in light cannabis smokers and patients with intractable epilepsy.^130^, ^131^ Additionally, an assay was developed to simultaneously quantify four mAbs and estimate Hct using haemoglobin peptides in DBS.^31^ The large size of mAbs restricts their movement through the cell membrane of erythrocytes causing an observable Hct effect; thus, this assay greatly benefits DBS‐based mAb studies. A study on home‐based use of VAMS by psoriasis patients treated with adalimumab revealed patients preferred VAMS to conventional sampling despite ∼50% being uncertain about the reliability of the sampling technique, reflecting the knowledge gap.^110^

### 3.3 Omics‐based biomarker research

Microsampling technologies have been evaluated in biomarker research studies for the diagnosis and prognosis of various conditions and the prediction and monitoring of response to an intervention.^24^, ^41^, ^42^, ^132^, ^133^, ^134^, ^135^, ^136^, ^137^, ^138^, ^139^, ^140^, ^141^, ^142^, ^143^, ^144^ Omics‐based approaches, such as metabolomics and proteomics, are increasingly employed for the generation of large amounts of data to facilitate the rapid discovery of biomarkers. A study assessed the DBS and DPS technology for non‐targeted lipidomic analysis as high throughput profiling and quantification of lipids could expand knowledge of underlying disease pathologies. The study's validated assay was capable of annotating 498 compounds covering 24 lipid sub‐classes. DPS showed excellent correlation (*R* ^2^ = 0.9851) to wet plasma, whereas DBS required an Hct correction. However, only 60% of compounds were found to be stable at room temperature after 28 days.^132^ A review of DBS in lipidomics concluded that DBS lipidome by MS/MS analysis could prove to be just as effective as traditional lipidome if DBS stability and extraction are fully optimised taking into account pre‐analytical factors such as storage, stabilisers, type of DBS filter paper and Hct.^133^ The VAMS technology was successfully implemented in a proteomics study to understand the pathophysiology of takotsubo syndrome. The analysis of 10 µL VAMS samples obtained from 80 patients revealed persistent differential protein expression between patients and controls, identifying potential therapeutic targets for recurrence.^134^ VAMS is also a viable microsampling technique for large‐scale precision medicine studies using archival frozen blood cell pellets as the detection and robust quantitation of up to 1600 proteins from a single VAMS shotgun analysis demonstrated a higher proteomic depth than a conventional shotgun analysis of undepleted plasma, which only allowed for the quantification of ∼324 proteins.^135^ Additionally, VAMS was used to assess the physiological changes associated with the level of training and performance of trail runners which revealed that the fittest trail runners had a better adaptation of bioenergetic pathways.^136^

A novel LC‐MS/MS assay measured endogenous uracil levels in TASSO‐SST samples to diagnose dihydropyrimidine dehydrogenase deficiency. The study showed no systematic or proportional bias between TASSO‐SST serum and venous plasma uracil levels. However, at‐home sampling using TASSO‐SST for uracil measurements is not feasible due to the compound's high instability. Nevertheless, microsampling technology can be implemented at the hospital to reduce the burden of repeated blood draws in cancer patients.^137^ A randomised, control trial evaluated the accuracy, feasibility and acceptability of microsampling techniques in rheumatoid arthritis patients by measuring inflammation markers and autoantibodies in three matrices: conventional venous blood, upper arm capillary blood obtained using TASSO‐SST and finger prick capillary blood. The upper arm and finger prick samples provided comparable results to venous blood samples. Based on a Net Promotor Score, upper arm sampling was found to be preferred over finger prick sampling by patients.^24^ Other studies that have implemented the VAMS technology include research for (i) the human biomonitoring of mercury concentrations for exposure assessment,^41^, ^145^ (ii) the determination of tryptophan‐related biomarkers for understanding pathologies of neurodegenerative diseases such as Alzheimer's or Parkinson's disease^138^ and (iii) the measurement of endocrine and bone biomarkers.^139^ VAMS technology was also capable of detecting carcinogens, such as acrylamide and aflatoxin B_1_.^140^, ^141^

The incidence of the global COVID‐19 pandemic has accelerated the use and development of decentralised blood sampling for virtual clinical trials, trials that do not require in‐person visits and routine analysis. The use of TASSO‐SST devices was evaluated for measuring blood protein levels in healthy subjects and non‐hospitalised COVID‐19 patients. The measurements of 17 protein inflammatory biomarkers using a multi‐analyte immune‐assay revealed that protein concentrations were higher in COVID patients compared to controls. However, a bias between TASSO‐SST samples and venous samples was found to differ significantly in D‐dimer, IL‐1B and IL‐1Ra, which could be attributed to differences in user proficiency, temperature control and the time lag between sample collection and processing.^42^ Microsampling has also been used for the serological analysis of SARS‐CoV‐2 infection in three studies.^142^, ^143^, ^144^

### 3.4 Forensic toxicology

Forensic toxicology analyses biological samples to determine drug usage, poisoning or exposure to toxic substances for use in a legal investigation. Here, isolation and identification of substances associated with criminal activity has primarily focused on blood samples. Antemortem or post‐mortem blood for forensic drug testing or post‐mortem toxicology must be appropriately sampled and adequately preserved to ensure the stability of compounds of interest which can have a profound impact on the interpretation of results and the outcome of forensic casework. Additionally, timely collection of biological evidence is a critical component in circumstances where drugs have limited detection windows or collection of post‐mortem samples becomes challenging as a result of autolytic and putrefactive changes.^146^ Thus, microsampling offers several advantages in forensic toxicological screenings for the analysis of drug abuse: (i) small sample size is beneficial when there is a limited sample, (ii) reduced exposure risk to blood‐borne pathogens ensuring the safety of personnel involved in sample collection and analysis and (iii) potential for faster, efficient and economical sample collection and long‐term storage with increased stability permitting accurate analysis and re‐analysis, even years after sample collection.^119^

One of the routine forensic toxicology practices is the assessment of alcohol intoxication using the blood alcohol concentration in cases of driving under the influence or traffic incident deaths. However, due to the short half‐life of ethanol in blood, typically between 4 and 5 h, other markers are evaluated to determine drinking behaviours and patterns.^147^ Phosphatidylethanol (PEth) is a unique direct ethanol biomarker with a broad detection window to determine alcohol consumption or abstinence. PEth 16:0/18:1, the most prominent homologue in human blood, can act as a complement to the ethyl glucuronide, a biomarker in urine, to distinguish alcohol abusers from social drinkers.^148^ In the year 2022, several research articles focused on the analysis of PEth 16:0/18:1 from microsamples acquired via DBS or VAMS technology. Two analytical methods were developed to quantify PEth in DBS samples, where the detection of PEth was achieved using UHPLC‐QTOF‐HRMS, UHPLC‐MS/MS and Orbitrap‐based HRMS.^148^, ^149^ In a study quantifying DBS PEth using an LC‐MS/MS method, the threshold of PEth for excessive alcohol consumption was determined to be 160 ng/mL when the blood alcohol concentration decision limit was 50 mg/dL. This threshold provided a sensitivity of 92% and a specificity of 90% for predicting excessive alcohol consumption.^35^ In contrast, a threshold below 20 ng/mL is associated with determining alcohol abstinence. However, given the 4–7 days half‐life of PEth, it can take months for PEth values to fall below this threshold following cessation of alcohol consumption. This can make PEth interpretation challenging, particularly when monitoring individuals with alcohol‐use disorders. To address this challenge, a population‐based algorithm was developed to estimate the cessation of alcohol use based on PEth values acquired from the analysis of VAMS samples obtained at three time points. The decision tree yielded a sensitivity and specificity of 89% by examining the relationship between two successive PEth values that were not yet below the decision limit for alcohol abstinence.^36^ Although single PEth determinations are beneficial in various forensic contexts, the added value of simultaneously determining three direct ethanol biomarkers was determined: 2 PEth isoforms (PEth 18:1 and PEth 20:4) and ethyl glucuronide in dried blood microsamples by evaluating several forensic cases.^150^ Furthermore, it was demonstrated that the distribution of seven different PEth homologues quantified in DBS can aid in better comprehending an individual's drinking history. The study also revealed the risks of sample contamination associated with microsampling for PEth analysis when excessively using ethanol‐based hand sanitizers.^151^

A major challenge in forensic toxicology is the analysis of substances of abuse due to the constant influx of new psychoactive substances (NPS), such as synthetic cannabinoids and opioids, cathinones and hallucinogens, which pose an unprecedented threat to public health. Recently, a UHPLC‐HRMS method was developed and validated for the accurate quantification of 132 NPS within an adequate concentration range of 5–100 ng/mL in 30 µL DBS samples. Most drugs analysed were found to be stable at 4°C for up to 40 days with extremely limited degradation. The method was applied to seven real samples, which provided quantitative results consistent with those obtained using conventional venous whole‐blood samples.^34^ Similarly, cocaine and its metabolites, benzoylecgonine and cocaethylene, were found to be stable for 7 days when DBS samples were retained within the HemaPEN® device.^152^ An LC‐MS/MS method was also established to detect 425 drugs, including several sedative hypnotics, antileptic drugs and antipsychotic drugs, in 10‐mm DBS punch samples. The method was successfully applied to samples obtained from actual drug‐poisoning cases that were stored for 3–5 years at room temperature. The results were in accordance with those measured at the time of sample collection except for a few compounds such as ambroxol, zopiclone, carbofuran and valproic acid, which were not detectable in the long‐term preserved DBS samples.^153^ As an alternative to DBS analysis, an HRMS method was developed to screen for 85 licit and illicit drugs in 20 µL VAMS samples, which showed a comparable distribution of substances between capillary and peripheral streams (*R* ^2^ = 0.9997).^154^

Microsampling was also used in the high‐throughput identification of toxins and trace elements, where a highly sensitive LC‐MS/MS method was developed for the measurement of ricinine and L‐abrine in DBS, with respective detection limits of 50 and 100 pg/mL. The method's complete analytical time was less than 1 h, allowing for rapid preliminary confirmation of the presence of highly toxic proteins, ricin and abrin.^155^ Although dried blood microsamples have increased stability for a variety of analytes, the authors showed that DBS performed poorly when quantifying trace elements such as aluminium, cadmium, cobalt and chromium, due to high signal contributions from blank filter paper and instability at room temperature. This study demonstrated that microtubes were more suited than DBS for trace element quantification in human blood.^156^

### 3.5 Sports anti‐doping

Microsampling has emerged as a valuable sampling technique for sports anti‐doping analysis as it reduces discomfort for athletes and allows for more frequent testing. These potential benefits have also been recognised by the World Anti‐Doping Agency (WADA) which has led to the legitimization of DBS for doping control in sports, including the Olympics. Several studies have evaluated the use of microsampling for the detection of prohibited substances, such as anabolic agents, growth factors and hormone and metabolic modulators. A recent critical review by Thevis et al. described the advances, potential future perspectives and limitations of blood microsamples in doping controls.^157^ Fast and cost‐effective screening methods to detect erythropoietin receptor agonists such as CERA, BRP, NESP and EPO‐Fc using dried blood microsamples were developed and optimised.^37^, ^38^ In the study conducted by Heiland et al., different blood sample volumes (20–180 µL) and storage temperatures (−20 to 37°C) were evaluated to determine optimal conditions for detecting prohibited ERAs from DBS. It was discovered that a blood volume of 60 µL allowed the detection of ERAs at levels comparable to WADA's minimum required performance levels (MRPLs) described for 500 µL of serum or plasma.^37^ In another study, CERA was demonstrated to be detected in DBS using a simple enzyme‐linked immunosorbent assay with a screening time of just 2 h.^38^ Furthermore, a multi‐analyte assay was developed and the performance of several non‐volumetric (Whatman cards‐Treated: Whatman FTA DMPK‐A, Whatman FTA DMPK‐B; Untreated: Whatman FTA DMPK‐C, Whatman 903) and volumetric devices (HemaSpot^TM^ HF, Mitra and TASSO‐M20) was evaluated for the simultaneous analysis of 235 drugs for doping control.^39^ Treated Whatman cards were found to produce very low recoveries or dirty extracts, whereas all other devices produced high recoveries between 60% and 90%. The LODs of the analytes ranged from 0.1 to 3.0 ng/mL, allowing for the detection of illicit use as most analytes were found to be detectable below the WADA's stipulated MRPL for urine. In addition, a fully automated DBS sample preparation and detection method was also developed for 13 anabolic steroid esters, frequently detected in sports doping controls.^158^

## 4 Summary and outlook

The advent of microsampling has radically transformed the landscape of blood sampling for various biomedical applications. The continued interest in microsampling has been driven by factors including minimal invasiveness of the approach, decentralised monitoring, logistical benefits, reduced biohazard risk, analytical improvements, data‐rich pre‐clinical and clinical studies and personalised health monitoring and treatment opportunities. Various microsampling technologies exist for the collection of dried or liquid matrix microsamples. DBS, the oldest microsampling technology, has continuously evolved over the years from a simple, Hct‐dependent, non‐volumetric collection on filter card to innovative devices for Hct‐independent volumetric collection mitigating its limitations of sample heterogeneity and volume bias. Modifications to the inherent filter paper component of DBS have the potential to provide additional functionalities, such as instantaneous proteolysis or stabilization. However, DBS is associated with Hct‐based recovery biases that necessitate correction factors, especially for analytes with high plasma‐to‐RBC partitioning ratio. This bias can be overcome with the use of DPS/DSS which is obtained by filtration of cells through passive separation membranes. This technology inherently eliminates the need for elaborate sample preparation as required in the analysis of whole‐blood samples and enables the direct comparison with plasma assays. These 2D dried matrix spots are susceptible to oxidative stress resulting from exposure to ambient air, rendering them unsuitable for the analysis of labile compounds. This vulnerability can be eliminated by utilizing the dried blood spheroid technology, which is a 3D blood collection technique that enhances sample stability by reducing the blood's exposed surface area to air. Alternative to paper‐based technologies, VTM produces volumetric dried blood microsamples independent of the Hct using polymeric tips. The dried matrix microsample collection typically involves a finger prick by lancet which enables the collection of 2–80 µL of blood. For collection of larger blood volumes (up to 600 µL), liquid matrix microsamples are collected either using capillary‐based or tube‐based technologies.

In recent years, the demand for microsampling devices has increased significantly in clinical research and diagnostic applications. This demand has led to a highly competitive market in which manufacturers continuously develop new, innovative and improved devices to offer enhanced performance, greater stability and more user‐friendly designs. Currently, the vast majority of commercially‐available devices require a simple finger prick by lancet to collect blood onto filter papers, capillaries or volumetric polymer tips. Continuous advancements in lancing device design, such as adjustable depth settings to accommodate varying skin thicknesses and retractable lancets to prevent needle stick injuries, allow for less painful, more efficient and more convenient blood sampling. Self‐sampling is particularly beneficial for individuals who need to regularly monitor their health, or who have difficulty with traditional blood collection, such as young children, the elderly or those with medical conditions that make blood collection challenging. Despite the ease of a finger prick in DBS, CMS and VAMS devices, the application of blood droplets onto the pre‐printed circle on the filter paper, application surfaces and tips, or their introduction into capillaries, requires manual interventions. These interventions can introduce manual errors and variability, thereby reducing sample quality. Further, CMS and VAMS devices may necessitate a higher level of technical expertise for accurate sampling. In addition, to generate enough volume of blood, lancing is often accompanied by milking of the finger which might result in haemolysis or an increase in the percentage of interstitial fluid collected, affecting downstream measurements. In the future, this problem could potentially be overcome with the use of hollow microneedles to sample blood by generating vacuum. Alternatively, vacuum‐assisted technologies to automatically collect blood from upper arm, such as the TASSO and TAP II devices, offer a simple self‐sampling design that deploys a lancet/microneedle array and enables automatic blood collection into tubes with the press of a button. In addition to self‐sampling capabilities, automation is a desirable feature of microsampling devices because it can significantly enhance the efficiency, accuracy and precision of sample analysis. Several automated DBS sample analysers have been developed to streamline sample analysis and reduce the time and effort required for sample preparation, processing and analysis. These analysers can handle a large number of samples quickly and accurately, making them ideal for high‐throughput applications. Similarly, the design of the Mitra samplers is suitable for extraction in microtiter plates, which can readily be further automated with minimal efforts.

Microsampling has been widely employed in different biomedical applications including animal studies, neonatal and paediatric applications, TDM, omics‐based biomarker research and forensic toxicology. Microsampling not only reduces the number of animals needed for studies in accordance with the 3Rs principle, but also improves data quality and animal welfare. It reduces the physical and psychological burden of blood sample collection from vulnerable populations such as neonates and young children. With the recent increased focus on personalised healthcare, microsampling provides opportunities for optimizing drug dosages through PK‐PD studies and TDM by enabling frequent sampling for data‐rich analysis. Longitudinal sampling and sampling during clinical episodes permit the collection of larger datasets for understanding disease biology and identification of diagnostic and prognostic biomarkers. The convenience and accessibility of at‐home self‐sampling increases patient compliance, diversity and recruitment in clinical trials. In addition, it allows for the secure and efficient collection of samples on‐site for forensic toxicology screenings. Several bioanalytical methods have been developed for the accurate quantification of analytes in microsamples using LC‐MS/MS analysis.

Despite the profound advantages over conventional sampling, widespread implementation of microsampling in clinical practice has been slow. While several factors contribute to this, a crucial factor is the quality of the samples collected. Implementing at‐home self‐sampling requires well‐defined user instructions on proper sample collection and adherence as well as strategies to monitor patient discipline to ensure sample quality and reduce data variability. Although dried blood microsamples can be shipped at ambient temperatures, extensive evaluation on stability must be performed for the analyte of interest to determine conditions for transportation and storage. Furthermore, optimization of the extraction method is essential in preventing reduced and inconsistent recoveries. Another significant barrier is the perspective of regulatory bodies on patient‐centric sampling approaches, which requires bridging studies to be performed to support the introduction of new matrices. Conventional matrices include venous blood, plasma or serum, whereas microsampling provides capillary blood. This inherent difference in sample type and composition can impact concentration of analytes; therefore, method validations and correction factors are necessary to produce reliable data comparable to conventional data. In addition, regular proficiency testing must be conducted to assure the quality, comparability and acceptability of analytical results, as there is no consensus over downstream processing protocols. And, while microsampling minimises the cost of storage, sample transportation and patient travel, the cost‐effectiveness must factor in the cost of the microsampling device and its potential limit of being a single‐use device.

Microsampling technologies can enable fully automated workflows to provide high‐throughput clinical testing. They can also be used in point‐of‐care devices to either facilitate improved patient care through at‐home testing and screenings or aid the development of wearable sensor‐enabled technology for continuous health monitoring, leading to digital transformation in healthcare and improved data connectivity. This review demonstrates that considerable advances have been made in recent times; however, data reported for many analytes are inconsistent across microsampling technologies, and in some cases, even for the same technology. Therefore, a continued effort is needed, including standardization, optimization of extraction process, validation of bridging studies and performance evaluation of technologies, for microsampling to be successfully implemented in routine clinical care to reap the benefits of capillary blood microsamples.

## AUTHOR CONTRIBUTIONS

Manchu Umarani Thangavelu: Conceptualization (equal); Data curation (lead); Writing – original draft (lead); Writing – review and editing (equal); Bert Wouters: Conceptualization (equal); Writing – review and editing (equal); Alida Kindt: Conceptualization (supporting); Writing – review and editing (equal); Irwin K. M. Reiss: Conceptualization (supporting); Writing – review and editing (supporting); Thomas Hankemeier: Conceptualization (supporting); Writing – review and editing (supporting).

## CONFLICT OF INTEREST STATEMENT

The authors have declared no conflict of interest.

## Data Availability

The data that support the findings of this review article are obtained from publicly available sources, including online databases such as PubMed and Google Scholar. Data sharing is not applicable to this article as no new data were created or analysed for the review.
